# Severe drug eruption from oral terbinafine for mild onychomycosis—A case report from family practice and literature review: “Just an innocent little pill?”

**DOI:** 10.1177/2050313X241235823

**Published:** 2024-03-04

**Authors:** Roeland M Watjer, Just AH Eekhof, Koen D Quint, Mattijs E Numans, Tobias N Bonten

**Affiliations:** 1 Department of Public Health and Primary Care, Leiden University Medical Center (LUMC), Leiden, The Netherlands; 2 Department of Dermatology, Leiden University Medical Center (LUMC), Leiden, The Netherlands; 3 Department of Dermatology, Roosevelt Clinic, Leiden, The Netherlands; 4 Department of Medical Microbiology, Leiden University Medical Center (LUMC), Leiden, The Netherlands

**Keywords:** onychomycosis, terbinafine, drug eruption, drug-related side effects, case reports

## Abstract

Onychomycosis is the most prevalent nail disease and is frequently encountered in clinical practice. Despite having multiple therapeutic options, of which systemic antifungals are the most effective, treatment is not always mandatory in all patients. Especially when considering systemic treatment, the risk of adverse reactions may outweigh the potential benefits of treatment. In this case report, we present a clinical case of a 49-year-old male patient with a blank past medical history who experienced a severe drug eruption from terbinafine prescribed for mild onychomycosis that required discontinuation of terbinafine, additional evaluation, and treatment of this adverse reaction.

## Introduction

Onychomycosis, a fungal infection of the nail caused by dermatophytes, yeasts, and non-dermatophyte molds, is the most common nail disease with an estimated prevalence of 4.3% in North America and Europe and a worldwide estimated prevalence of 5.5%, respectively.^1,2^ However, the prevalence of onychomycosis can increase markedly with advancing age, with underlying conditions such as diabetes, or in different continents and climates.^3–6^ Although frequently having an indolent course, onychomycosis is considered not to resolve spontaneously.^7–10^ Onychomycosis severity can range from mild, affecting only a limited portion of the nail, to severe, involving the majority of the nail plate and the nail matrix, and/or causing substantial subungual hyperkeratosis and dermatophytomas.^11^ Regardless of severity, many patients find the associated changes disfiguring and bothersome, and in a considerable number of patients, onychomycosis can affect the quality of life.^12–15^ By contrast, onychomycosis remains asymptomatic in many patients, often unaware of having onychomycosis in the first place.^2,3,6,16,17^

Not only those suffering from evident symptoms or complications but also those with predominantly cosmetic concerns will consult their physician. Requests for treatment are not seldom driven by commercial campaigns that draw attention to onychomycosis and frame it as ugly, unhygienic, and a disease that requires treatment.^18^ However, according to current guidelines, treatment is not always mandatory.^19,20^ Physicians may suffice by providing the necessary information and, if applicable, reassurance. Satisfactory for some, other patients may persist in their request. If treatment is indicated, oral terbinafine for a minimum of 3 months is recommended.^19–22^ In terms of cure rates, terbinafine achieves 70% mycological, and 38% complete cure, the latter consisting of both clinical and mycological cure.^23^ Despite being the most effective option, a 38% chance of a complete cure could arguably be regarded as modest; at the end of the day, patients will primarily be interested in normal-looking nails after completing their treatment. Even after successful treatment with terbinafine, a recurrence rate of 33% after an average of 36 months is significant.^21,24^ Moreover, terbinafine can have potentially serious side effects, such as severe skin reactions, and cases of liver failure have been described.^25–27^

In this case report, we present a case of a patient who developed a severe adverse reaction to terbinafine, illustrating an important potential harm of oral antifungal treatment prescribed for onychomycosis. Written informed consent was received on July 30th, 2023.

## Case description

**Figure 1.**
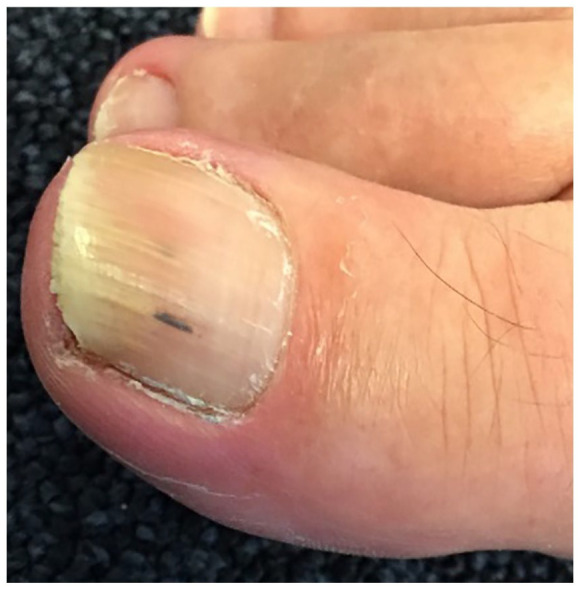
The nail as presented before starting terbinafine.

A 49-year-old male patient with a blank medical history visited our practice because of a discolored greater toenail. Normally rarely attending the clinic, he was embarrassed about his abnormal-looking nails. Having tried multiple home remedies, he decided to make an appointment. He explained that at first, only the greater toenail was affected. Now, it seemed to be spread to adjacent nails which disconcerted him. After reading information on the internet, he opted for oral terbinafine. There were no mechanical issues, pain, or concomitant infections present. On examination, his toenail was moderately affected (Figure 1). Given the patient’s preference and after explaining the expected results and potential side effects, the attending physician provided the requested prescription.

**Figure 2.**
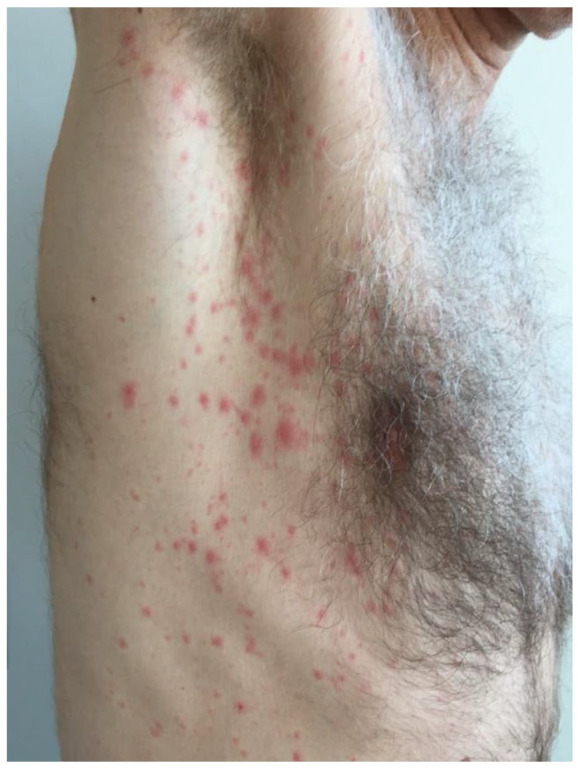
The skin reaction after 11 days.

**Figure 3.**
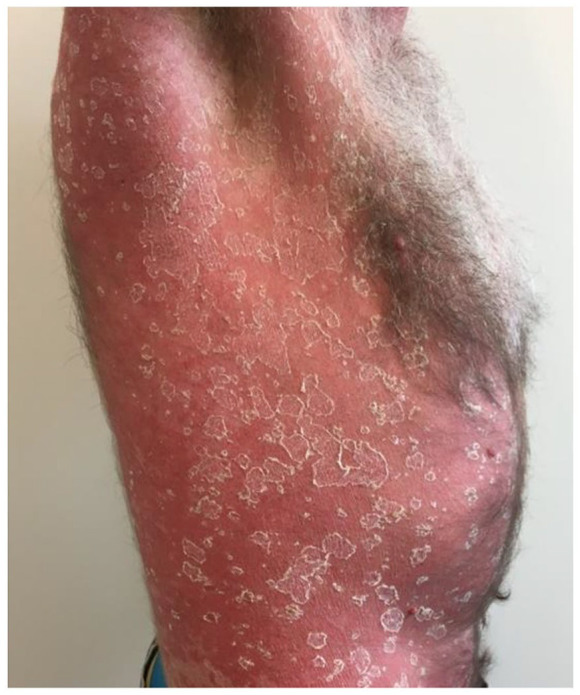
The skin reaction after 15 days.

Eleven days later, he returned to the clinic with a red, non-itching macular rash on his chest and axillary region (Figure 2). Suspecting a mild allergic reaction, fexofenadine 180 mg o.d. was prescribed, and terbinafine was discontinued. Four days later, fexofenadine not having improved his symptoms, he returned to the practice again. His upper body had turned “completely red” (Figure 3). On examination, a confluence erythematosquamous rash was seen, suggestive of exfoliative dermatitis.^28^ A severe allergic reaction to terbinafine was suspected, and after consulting a dermatologist, prednisolone 30 mg o.d. was started, and the patient was referred to the dermatologist.

On examination, we observed an erythrodermic patient with a diffuse erythematosquamous rash with collarette-shaped desquamations, extending from the neck over the entire torso, to the arms and thighs. The lower legs and hands were showing smaller erythematosquamous papules. No bullae or vesicles were present. The oral mucosa and eyes were not affected, and his temperature was normal. To exclude a drug reaction with eosinophilia and systemic symptoms (DRESS syndrome), a blood test was performed, showing an elevated erythrocyte sedimentation rate (ESR) (25 mm/h), leukocytosis (27.8 × 10^9^/L), but no eosinophilia or other blood count abnormalities, nor any abnormal liver or kidney function tests. Thus, the dermatologist’s conclusion was a severe drug eruption from oral terbinafine in the form of exfoliative dermatitis, confirming our suspected diagnosis. The consulting dermatologist decided not to perform a skin biopsy due to the highly suggestive history and findings on physical examination. Prednisolone was continued, and topical treatments in the form of betamethasone, Calmurid, and vaseline paraffin ointment were added. Three weeks later, returning to our practice for follow-up, the rash was fully resolved and the skin had returned to normal.

## Discussion

### Case evaluation

This case represents an example of a severe drug reaction to terbinafine, a relatively rare but well-known adverse event.^29^ Although our patient was very much bothered by the cosmetic changes caused by the fungal infection, the onychomycosis was mild and without additional consequences or complications that otherwise would have warranted treatment. Oral terbinafine provided an opportunity to rid this patient of his onychomycosis but also exposed him to potential adverse reactions, as illustrated.

### Review of the literature

As for most antifungals, the primary mechanism of action of terbinafine is the inhibition of fungal membrane production and ergosterol synthesis.^30,31^ Terbinafine belongs to the group of allylamines and works as a non-competitive inhibitor of the enzyme squalene epoxidase, preventing the conversion of squalene to squalene-epoxide.^8^ The most common adverse reactions are headaches, gastrointestinal symptoms, and rashes.^31^ Less common adverse reactions include visual disturbances, dysgeusia, and transient transaminitis.^31^ Regarding the skin, rashes not otherwise specified (6%), pruritus (3%), and urticaria (1%) are most frequently reported.^32^ Specific cutaneous conditions linked to terbinafine use include fixed drug eruption, erythema multiforme, erythema annulare centrifugum-like eruption, sub-acute lupus erythematosus, flare-ups of psoriasis, psoriasis de novo, and more severe reactions such as exfoliative dermatitis, acute generalized exanthematous pustulosis, and toxic epidermal necrolysis.^32–34^ No specific incidence rates are available for specific skin reactions, including exfoliative dermatitis as in our case.^32^ Although most adverse reactions to terbinafine are mild and do not warrant discontinuation, some are more severe, and even cases of fulminant liver failure requiring liver transplantation have been reported.^35^ However, as for adverse skin reactions, incidence rates for specific hepatic reactions are lacking, and only rough estimates are mentioned, for example, “0.01%” or “less than 1 in 1000.” ^8,32,36,37^ Regarding alternative treatments for onychomycosis as opposed to oral terbinafine, other systemic antifungals or topical treatments might have been considered. However, alternative systemic antifungal treatments pose similar risks to adverse reactions, and topical treatments, although carrying a much lower risk of serious adverse reactions, are unfortunately significantly less effective than systemic antifungal treatment.^21,38^ Our case was an example of a rare but severe cutaneous adverse reaction to terbinafine in the form of exfoliative dermatitis that warranted the discontinuation of terbinafine and the addition of systemic corticosteroid treatment.

## Conclusion

Patients visiting their family physician often wish to receive a form of treatment, hoping this will resolve their problem. However, many problems presented do not require pharmacological treatment. Some issues cannot be solved at all, some are self-limiting in nature and only require symptomatic treatment, and some problems do not warrant treatments despite having multiple treatment options. A mild case of onychomycosis, without an increased risk of complications, is a good example of the latter, since treatment is not always warranted in such cases. Physicians, however, may be tempted to prescribe treatment to comply with the expectations of the patient, rather than making a choice primarily based on medical necessity or urgency. As for any treatment, physicians should weigh the severity, burden, and expected outcome of a disease against the potential harms of the treatment considered. Oral antifungal treatment for onychomycosis not only cures less than half of the patients treated, with onychomycosis recurring in one-third of patients but also has the potential to cause severe adverse reactions, as illustrated. The principle of “primum non nocere” incorporated in the Hippocratic oath and roughly translated to “abstain from all intentional wrong-doing and harm” still holds. When confronted with medical challenges and having to choose between treatment with an uncertain outcome or watchful waiting, the latter is often the better choice.
